# An Overview of Strategies and Response to the COVID-19 Pandemic in Vietnam

**DOI:** 10.2188/jea.JE20240181

**Published:** 2024-12-05

**Authors:** Truong Son Nguyen

**Affiliations:** 1 Ministry of Health Vietnam, Hanoi, Vietnam; 2 Cho Ray Hospital, Hochiminh, Vietnam

Coronavirus disease 2019 (COVID-19) was declared a pandemic in March 2020, causing a global spread of the novel severe acute respiratory syndrome coronavirus 2 (SARS-CoV-2) virus and a public health emergency.^1^^,^^2^ Despite being a low–middle-income country with limited resources, Vietnam managed to control the virus spread and transitioned to a new normal on October 10, 2021. Our study aims to summarize the actions taken by the Vietnamese government and the Ministry of Health (MOH) to contain COVID-19 amidst several challenges.

This study collected main data from Jan 1, 2020, to Jan 31, 2022, from MOH (https://covid19.gov.vn) and the legal library (https://thuvienphapluat.vn/) and obtained relevant documents from official and reliable sources worldwide.^3^^,^^4^

Vietnam has been on high alert since December 2019 due to COVID-19. The government established a national strategy based on World Health Organization (WHO) guidelines. The National Steering Committee, led by the Minister board, is responsible for directing pandemic control and assessment on behalf of the government. They established COVID-19 prevention steering committees from province to commune levels. Within a month, 63 provincial and 707 district steering committees were established.^5^^–^^8^ Vietnam implemented a strategy to prevent COVID-19 spread that included early detection, contact tracing, unique area-specific lockdowns, and quarantine. Vietnam’s pandemic strategy has changed to adapt to the Delta variant by incorporating vaccination, self-testing, and a three-tier treatment system, moving away from their previous “Zero COVID” policy to a “safe adaptation, flexibility, and effective control of the COVID-19 pandemic”.^6^^,^^9^

The government communicated national strategies to society through various documents. In 2020–2021, they released seven directives, 20 decisions, 13 dispatches, four telegrams, and eight resolutions to coordinate and implement pandemic prevention measures.^3^^,^^4^

The MOH coordinates COVID-19 measures among its sub-agencies and ministries, following the National Response Plan based on WHO’s guidelines, updated response plans with 197 technical documents, and prioritized COVID-19 vaccinations guided by global updates.^4^^,^^6^^,^^10^

Vietnam’s healthcare system comprises central, provincial, district, and commune levels. It faces significant challenges in both public and private models, with a persistent shortage of healthcare workers. In response, the MOH has established a special unit led by the Vice Minister to mobilize and manage healthcare personnel. This unit develops strategies to control hotspot outbreaks directly using resources from hospitals and universities. More than 700 medical students and healthcare workers were deployed in response to the outbreak in the northern region, and 14,543 volunteers were mobilized in the southern region in 2021.^11^^–^^15^

**Figure 1.**
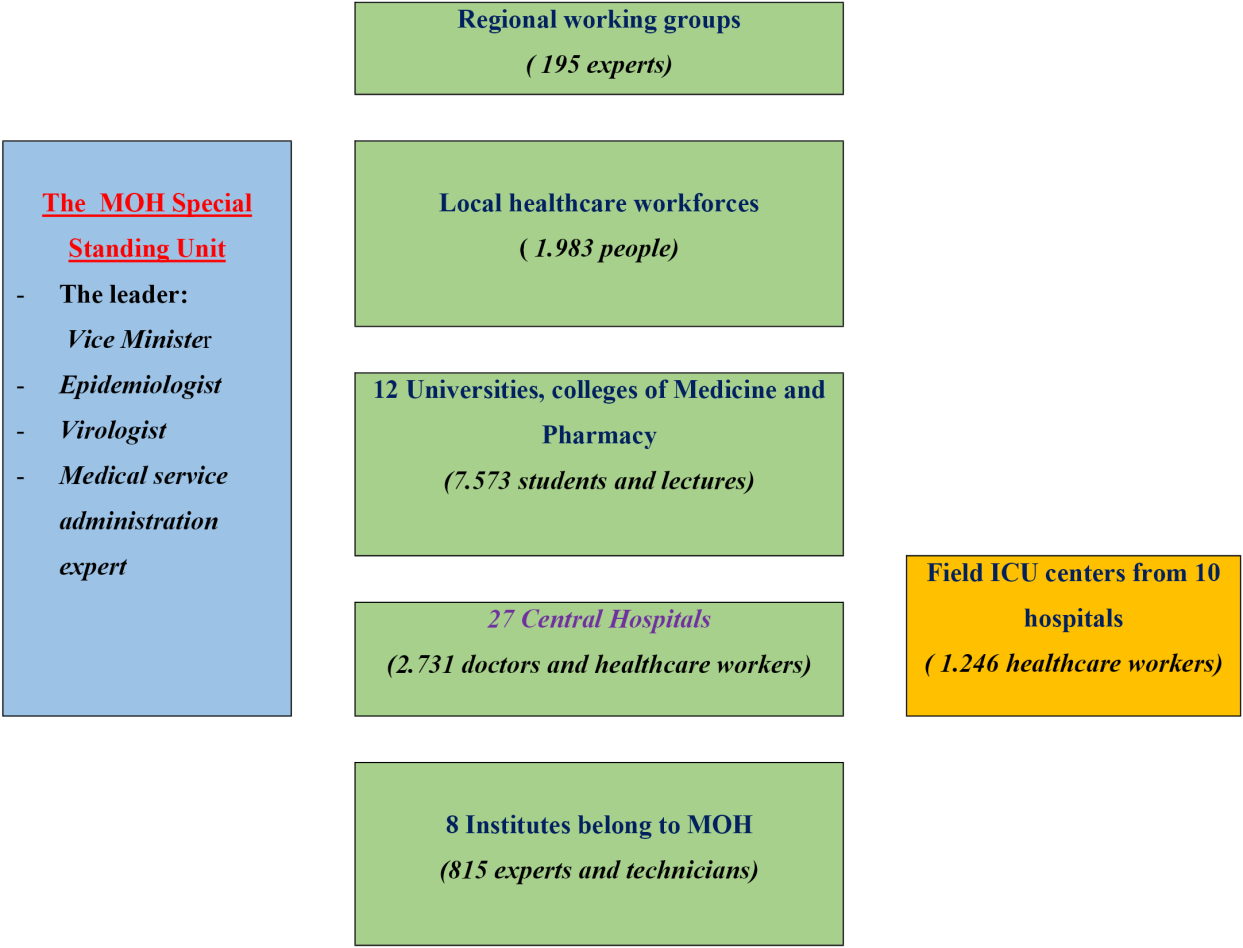
Flowchart for mobilizing and managing healthcare personnel in Southern Vietnam during the fourth surge of COVID-19 in August 2021. COVID-19, coronavirus disease 2019; ICU, intensive care unit; MOH, Ministry of Health.

**Figure 2.**
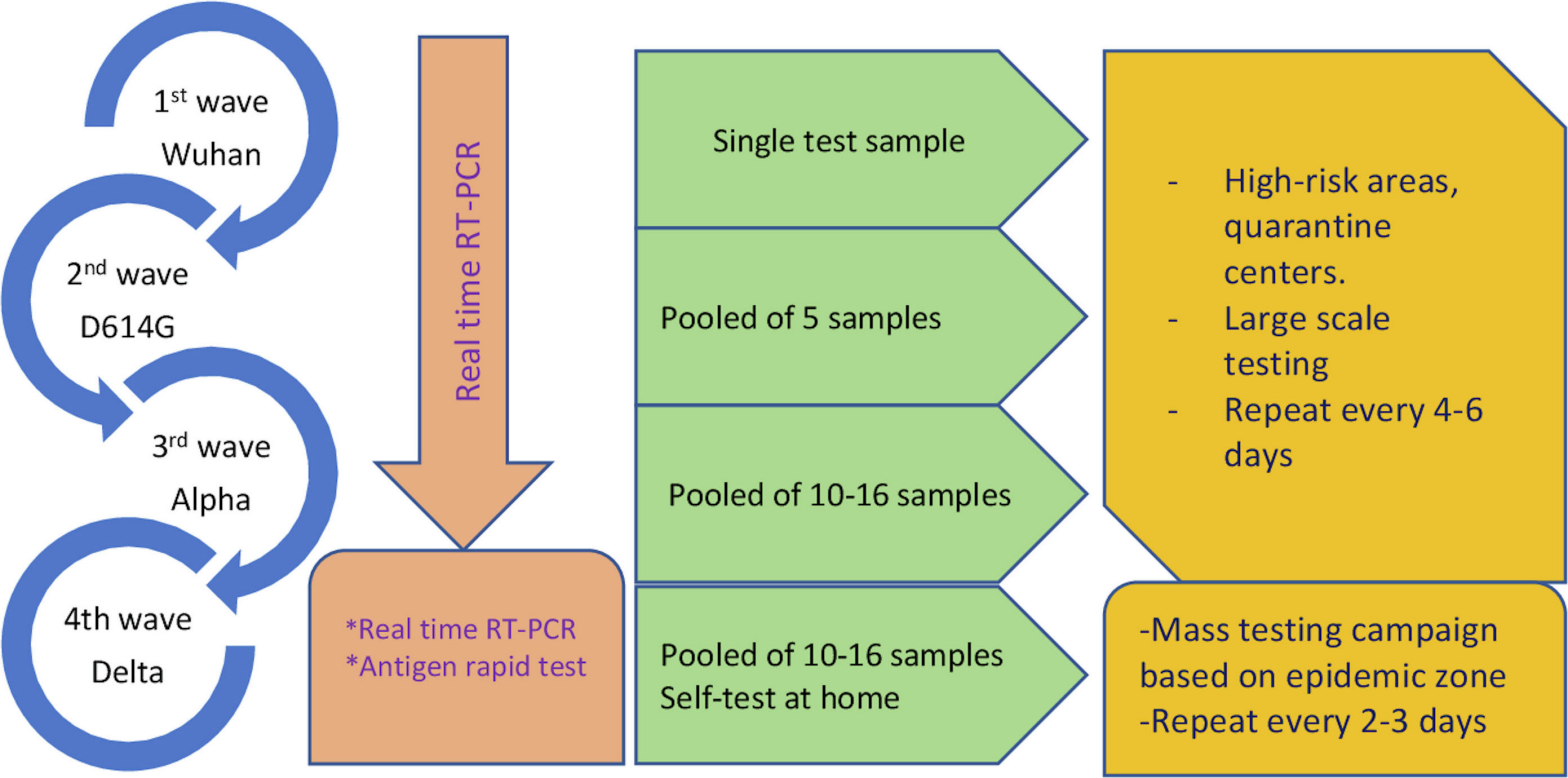
Diagram of testing strategies during the COVID-19 pandemic in Vietnam. COVID-19, coronavirus disease 2019; RT-PCR, reverse transcriptase polymerase chain reaction.

**Figure 3.**
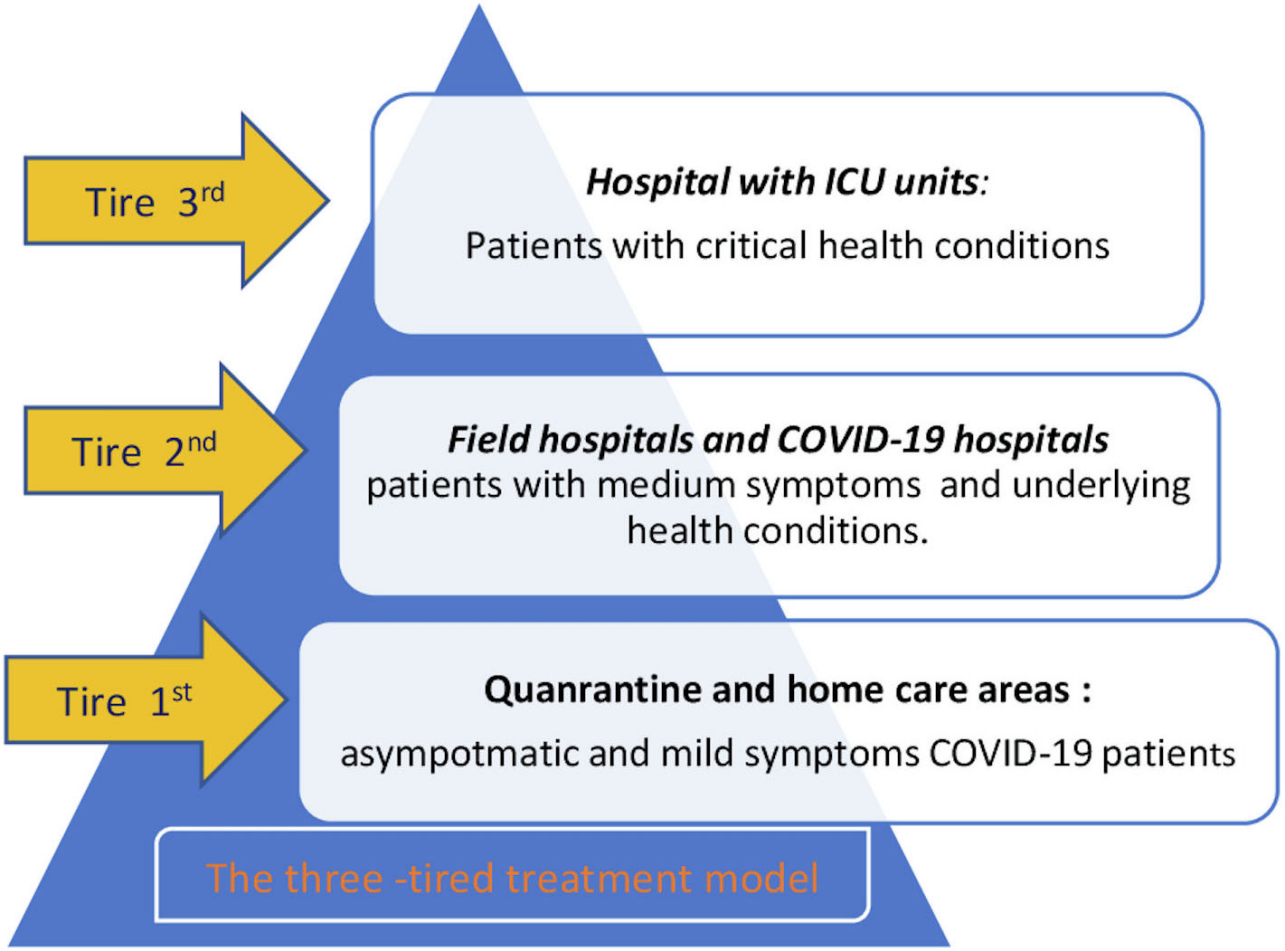
Diagram illustrating the three-tiered treatment model implemented during the fourth surge of COVID-19 in Vietnam. COVID-19, coronavirus disease 2019; ICU, intensive care unit.

Efficient testing, treatment, and vaccination strategies were designed to approximate available resources and support the public health objective of minimizing the death rate. The MOH provided guidelines for large-scale NAAT-PCR testing on targeted populations, utilizing various pooling tests and screening through self-administered rapid antigen tests. The MOH has implemented various measures to enhance the treatment process for COVID-19 patients using a three-tiered treatment model. A major solution is the implementation of mobile medical stations in local communities, which provide first aid, testing, vaccinations, and referrals and have allowed 80% of COVID-19 patients with mild or asymptomatic symptoms to receive treatment at home (Figure 1, Figure 2, and Figure 3).^6^^,^^11^^,^^16^^,^^17^

By implementing flexible strategies and involving society, Vietnam successfully combated the COVID-19 pandemic (Table 1). The mortality rate in Vietnam is 1.19%, lower than the global average of 1.94% and the Western Pacific region’s rate of 1.32%. As of December 26, 2021, Vietnam has one of the highest COVID-19 vaccination rates globally, administering approximately 148.28 vaccine shots per 100 people.^2^^,^^6^^,^^18^^,^^19^ Despite the pandemic affecting different countries in different ways, after more than 2 years, Vietnam has successfully combated the pandemic, minimizing its economic impact and enabling the country to return to normalcy.

**Table 1.** The milestone of the COVID-19 pandemic in Vietnam and the actions taken in response

Time duration	Outbreak surge	Virus variant	Strategy	Intervention measure priority	Outcomes
Jan 23^rd^ to Apr 16^th^, 2020	Wave 1	Wuhan	Prevention, detection, isolation, zoning, stamping out and effective treatment (***zero COVID***)	- Quarantine - Distancing - Contact tracing - Targeted lockdown	- Cases: 415 (96 local cases^a^; 319 imported cases^b^) - Death: 0
July 25^th^ to Dec 1^st^, 2020	Wave 2	D614G			- Cases: 1,136 (571 local cases; 565 imported cases) - Death: 35
Jan 28^th^ to Mar 25^th^, 2021	Wave 3	Alpha (B.1.1.7)		- Testing - Effective treatment - Immunization	- Cases: 1,305 (910 local cases; 395 imported cases) - Death: 0
Apr 27^th^ to October 10^th^, 2021	Wave 4	Delta (B.1.617.2)			- Case: 835,036 local cases - Death: 20,520
October 11^th^ to Dec 30^th^, 2021		Delta (B.1.617.2) Omicron (B.1.1529)	Flexible adaptation and effective control of the COVID-19 epidemic	- Immunization - Self -test at home - Effective treatment	NA^c^
The Government response		- Developed the National Steering Committee (NSC) - Issued 7 directives, 20 decisions, 13 dispatches, 4 telegrams, and 8 resolutions - The resolution No.128/ NQ-CP to temporarily regulate safe and flexible adaptation along with effective control of the COVID-19 pandemic (October 11, 2021)			
The Ministry of Health response		- Developed National Response Plan - Created and issued 197 technical guidelines - Established the Standing Special Unit at hotpots of outbreaks. - Mobilized and managed of healthcare personnel			

^a^Local case: A case of local transmission has been confirmed in the area.

^b^Imported case: A confirmed case has been detected in a quarantine center, who had arrived in Vietnam from an affected country.

^c^NA: not applicable.
